# “It’s coming whether we want it to or not”: A qualitative exploration of older adults’ comfort with and perceptions of technology and digital health

**DOI:** 10.21203/rs.3.rs-8001649/v1

**Published:** 2025-11-19

**Authors:** Anjali Vasavada, Lorella Palazzo, Casey Luce, Magali Sanchez, Matthew Triplette, James D Ralston, Lisa Carter-Bawa, Beverly B Green, Hongyuan Gao, Christopher I. Li, Melissa L Anderson, Yu-Ru Su, Kristine Rogers, Karen J. Wernli

**Affiliations:** University of Washington School of Public Health; Kaiser Permanente Washington Health Research Institute; Kaiser Permanente Washington Health Research Institute; University of Washington School of Public Health; Fred Hutchinson Cancer Center; Kaiser Permanente Washington Health Research Institute; Hackensack Meridian Health; University of Washington School of Public Health; Kaiser Permanente Washington Health Research Institute; University of Washington School of Public Health; Kaiser Permanente Washington Health Research Institute; Kaiser Permanente Washington Health Research Institute; Kaiser Permanente Washington Health Research Institute; Kaiser Permanente Washington Health Research Institute

**Keywords:** digital health, technology, older adults, lung cancer screening

## Abstract

**Background:**

Older adults bear a disproportionate cancer burden but remain underrepresented in digital health intervention trials compared to younger counterparts. Since the COVID-19 pandemic, engagement with telemedicine and patient portals through the electronic health record (EHR) has grown for all age groups, suggesting readiness to adopt digital health tools. This qualitative study primarily sought to understand how adults eligible for lung cancer screening (LCS) engage with technology and digital health in their daily lives. The secondary objective was to assess acceptability and compatibility of a video-based LCS health communication as a digital health tool.

**Methods:**

Semi-structured interviews were conducted with 15 participants aged 51–80 through videoconferencing or telephone. Transcripts were analyzed using a rapid team-based analysis approach. The Consolidated Framework for Implementation Research (CFIR) was used as a guiding framework from throughout the study, with constructs of interest informing interview guide questions in data collection, and CFIR-mapping to generate a code list in the analysis.

**Results:**

Our findings generated four CFIR-informed themes, with 8 subthemes: 1) Internal facilitators: comfort with technology, self-efficacy in troubleshooting; 2) External facilitators: leveraging internet for health information, use of wearable devices, patient portal functionalities; 3) Internal barriers: emotional response, social isolation; 4) External barriers: scamming and data privacy. When shown the LCS video-based health communication, participants described general approval of the content and delivery but expressed concerns about safety related to accessing the video due to its delivery via weblink.

**Conclusions:**

Broadly, we found that older adults had high levels of technology use and leveraged various digital tools (such as wearable devices, mobile applications, and EHR patient portals) to manage their health care needs. Our findings underscore that older adults are active users of digital tools, yet persistent concerns about privacy, social isolation, and emotional burden must be addressed for digital health interventions to be acceptable and sustainable in this population.

**Trial registration:**

ClinicalTrials.gov: NCT05747443; 2023-02-17

## Background

The risk of developing cancer increases as people age, with roughly 88% of cancer cases in the U.S. diagnosed in adults aged 50 and over [1]. Adults remain eligible for cancer screening until ages 75–80, depending on cancer type. In the last decade, digital health interventions, which include wearable devices (e.g. Fitbit^®^, Garmin^®^, Apple Watch^®^), mobile health applications, and telehealth [2], have been increasingly employed in various trial and observational studies to improve cancer screening uptake [3], treatment monitoring [4, 5], and rehabilitation [4]. However, despite the high proportion of older adults affected by cancer in the United States, older adults are routinely excluded from cancer screening trials testing digital health interventions relative to their younger counterparts, potentially attributed to ageism in healthcare [6].

Research conducted prior to 2019 has demonstrated lower use of digital health tools and technology among older adults compared with younger adults [7–9]. While empirical research is limited, some have speculated that lower use is related to older adults’ desire for in-person communication with their health care team, or due to rising concerns about privacy and security when using digital health technologies [9–11]. However, since the COVID-19 pandemic, older adults have increased their engagement with digital health through telemedicine visits and frequently communicate with their care teams through the electronic health record (EHR) patient portal [6, 12]. Additionally, qualitative studies including older adults suggest that this population is keen to learn more about technology and adapt to newer digital tools [13, 14].

This qualitative study primarily sought to understand how adults eligible for lung cancer screening (LCS) engage with technology and digital health in their daily lives. The LCS-eligible population is aged 50–80 [15], as such, understanding digital health and technology use in this population can provide a unique lens through which we can examine older adults’ use and comfort with technology. Lung cancer remains the leading cause of cancer death, with approximately 120,000 deaths attributed per year [15]. Despite the particularly high burden of lung cancer in older adults where median age at diagnosis is 71 years in the United States [15], minimal research is ongoing employing digital health interventions to increase screening [16, 17]. The secondary objective of this study was to assess acceptability and compatibility (i.e. perceived fit and benefit of the intervention) of a video-based LCS health communication as a digital health tool for the LCS-eligible population. Together, both objectives provide greater context and understanding of the preferences of older adults to inform creation of future digital health communication, both for LCS and cancer screening overall.

## Methods

### Study context and design

The qualitative study was embedded in the Larch Trial, a pragmatic clinical trial testing a digital health intervention and stepped-reminders to improve annual repeat LCS. The details and protocol for the trial are described elsewhere [18]. Trial participants were individuals empaneled with a primary care physician (PCP) from Kaiser Permanente Washington (KPWA), an integrated delivery system that provides insurance and serves ~ 600,000 members across Washington State [19]. Participants of the Larch trial were individuals eligible for LCS by U.S. Preventive Services Task Force guidelines [20] with a LCS low-dose computed tomography (LDCT) scan with normal or benign findings (LungRADS 1 or 2) performed during the enrollment period.

### Digital health intervention: Patient Voices Video

The *Patient Voices Video* (https://kpwashingtonresearch.org/kplung) was a health communication intervention that emphasizes the importance of returning for annual LCS for repeat screening. The video was delivered via secure message within electronic health record (EHR) patient portal, which includes a weblink to access the video. The development and design of the *Patient Voices Video* has been described previously [18]. Three key messages in the video were: a) Participating in LCS is an important step in taking care of your health by finding lung cancer early (i.e., normalize LCS); b) You are due for your next lung scan in 12 months (i.e., provide information when due); and c) Talking with friends and family about LCS might be helpful in feeling supported that you are doing something positive for your health (i.e., suggest social support).

### Study population

We conducted semi-structured interviews with 15 KPWA members who were excluded from the Larch Trial because they did not have an empaneled PCP, but otherwise LCS eligible by age and tobacco history, and completed a LCS LDCT within trial period. The population was selected to ensure that there was no bias in the interviews from receiving the digital health intervention as part of the trial, while ensuring that the sample interviewed broadly resembled the overall LCS-eligible population. We recruited a balance of participants aged 50–65 and 65 + to gather perceptions of individuals across the entire LCS age spectrum. The study was approved by the Kaiser Permanente Interregional IRB (Study #2139657).

### Recruitment

A study team member mailed recruitment letters and an information sheet to invite eligible patients to participate in a ~ 45 minute interview via Microsoft Teams or telephone call. The study team followed up with non-responders by telephone call up to two times. If potential participants did not respond after the initial mailing and two telephone calls, they were removed from follow up. If a potential participant expressed interest in completing an interview, a study team member sent an email to schedule a mutually convenient interview slot. If participants indicated interest in a Microsoft Teams meeting, the email contained a link to the Microsoft Teams call and generic instructions on how to use Microsoft Teams. If they indicated interest in a phone call, then the email included the date and time of the interview, and the interviewer called the participant at the agreed upon time. Participants provided oral consent to interview at the start of interview. Participants received a $75 cash incentive after completion of the interview.

### Guiding framework

The updated Consolidated Framework for Implementation Research (CFIR) was the guiding framework for the interview guide and code list [21]. The updated CFIR includes COM-B constructs [22], which allows for the comprehensive exploration of multiple constructs as they influence patient needs, capability, motivation, and preferences around digital health and technology, as well as barriers to use.

### Interview guide and procedures

The semi-structured interview guide was developed in consultation with the trial principal investigator (PI) and team members (Table 1). The guide was then piloted with study team members and a Larch Trial Patient Advisory Board (PAB) member. Feedback to the guide and overall interview flow was incorporated based on the pilot interviews. The interview guide included topics on participants’ general comfort level with technology, use of digital health, experience with the patient portal, and reactions to the *Patient Voices Video*.

The interviewer shared the *Patient Voices Video* with the participants via shared screen if the interview was conducted on Microsoft Teams. If on a telephone call, the interviewer walked the participant through accessing the video via weblink. All interviews were audio recorded and transcribed. After each interview, summaries were generated summarizing key points and insights to share with the research team.

### Data Analysis

Data analysis was done in two phases. In the first phase, the lead researcher (AV) developed a code list comprising of CFIR-mapped deductive codes and inductive codes generated from interview summaries. The second phase consisted of a rapid group analysis phase leveraging codes from Phase 1 to analyze interview transcripts to identify key themes and findings.

#### Phase 1: Code list development

A CFIR-mapping technique was employed to elicit an initial code list. This was done by mapping CFIR constructs to individual interview questions to develop deductive codes. Inductive codes were then generated by reviewing interview summaries and debriefs to create an overall code list with seven codes. These seven codes were employed as the analytic domains in Phase 2.

#### Phase 2: Rapid group analysis

We employed the Rapid Group Analysis Process (Rap-GAP) method for data analysis developed by Hsu et al [23]. Rap-GAP is a five-step rapid analysis method that relies on group-based coding and thematic analysis to analyze qualitative data. Rap-GAP was selected as the analysis method due to its efficiency, emphasis on primary data (e.g. transcripts), and collaborative nature.

In Step 1, the Rap-GAP lead (AV) identified 5 research team members (AV, LP, CL, MS, KJW) who all have qualitative research experience to participate in the group analysis. In Step 2, the Rap-GAP team members independently reviewed and coded 2–4 transcripts, documenting insights and illustrative quotes into an excel workbook that included the pre-developed code list as separate sheets. In Step 3, the Rap-GAP team lead uploaded all the coded insights to a virtual white board, which was used as the workspace for the Rap-GAP session. The 90-minute session consisted of a collaborative process where insights were grouped together within domains to generate emergent themes. In Step 4, the Rap-GAP lead exported all insights, quotes and themes from Steps 2 and 3 to create a master analytic dataset. Finally, in Step 5, the lead created a coding memo compiling all major findings and circulated it to the Rap-GAP participants for confirmation and validation (Table 2).

## Results

### Participant Characteristics

We sent mailings to 251 potentially eligible participants for interviews and conducted interviews with the first 15 eligible respondents to reach the target sample size. Sex was balanced (53% male, 47% female). The median age was 68 years, with a range of 51–75 years, which closely reflected the overall age range of individuals eligible for lung cancer screening. The sample was somewhat balanced across age groups, with 40% of participants aged 50–64, and 60% participants aged 65+.

### Themes

**Figure 1:**
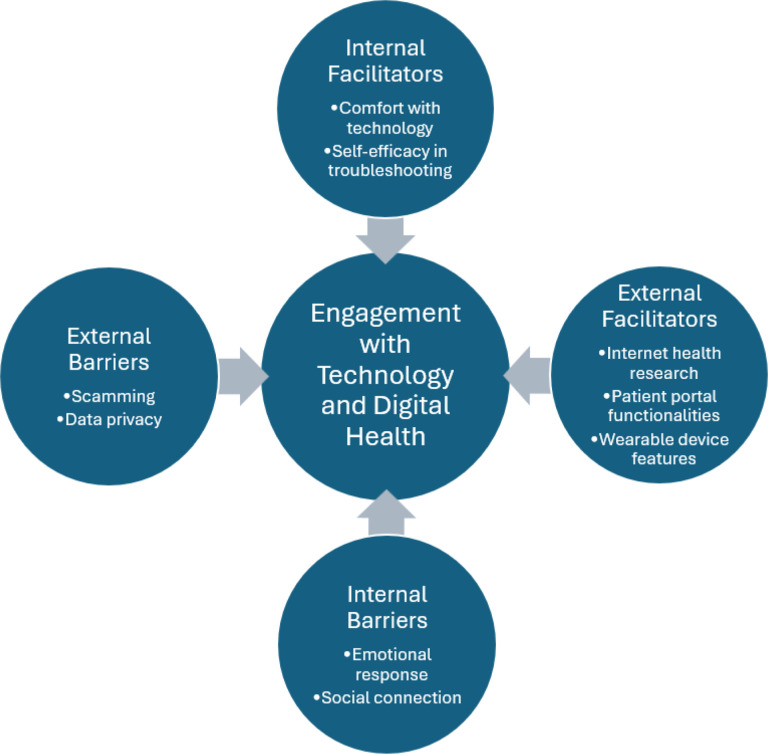
CFIR-Derived Themes

Our primary analysis elicited 8 themes, which were grouped under four CFIR-derived constructs: internal facilitators, external facilitators, internal barriers, and external barriers (Fig. 1). The secondary objective elicited information about the compatibility of the video intervention in the context of overall participant attitudes and perceptions of technology. Overall, our findings illustrate that older adults engage with technology in meaningful ways to improve their health but continue to have fears related to data privacy and security, which may impact their engagement with digital health interventions. The themes and associated illustrative quotes are described in detail below.

### Facilitators to technology and digital health use

#### Internal Facilitators

Internal facilitators of digital health use include factors that are intrinsic to participants that motivate them to use technology in a meaningful way. Generally, participants expressed comfort and confidence using technology in their daily lives. They used a variety of digital devices and mobile applications but specifically called out preference of smartphones over computers or tablets. Most participants described self-efficacy in troubleshooting technological challenges, leveraging Google or YouTube, or asking their children for support, if necessary.

##### Comfort with general technology use

Overall, most participants reported comfort using technology and described frequent use of different types of technology throughout their days, utilizing technology for different facets of their lives, ranging from work to entertainment.

I use it [technology] for everything. I’m probably more computer literate than most people my age. So I use my laptop for news all day long. I use it to find reading. You know the likes that I enjoy. I use it for paying all my bills. I use it for entertainment at night.

-Male, 69 years

I use technology when I get up in the morning. And I get on my phone and I play games, check my emails, check different social sites. So that’s pretty much my phone and then I get onto my computer and check other sites where I want to have a larger screen. I play crossword puzzles. I contact my kids through text.

-Female, 68 years

While participants used various forms of technology in their day-to-day lives, an overwhelming majority of participants expressed a preference for using their smartphones over computers.

I am addicted in that respect of having my phone. I used to be on my computer. I have a little Apple laptop that I used quite a bit but I’m not on it so much. My phone has taken over most of my computer stuff, I’d have to say.

-Female, 70 years

This is likely in part due to the high availability of the modern smartphone, but also because the smartphone often makes it easier to complete daily tasks, including communicating with medical providers.

It’s where I do most, I transact most of my kind of life things. Whether it’s banking, or conversing with my doctor, scheduling appointments with my haircutter, making reservations. So I’m a pretty avid app user. I would, I would say.

-Male, 51 years

##### Self-efficacy in troubleshooting technology issues

When faced with any technological issues, participants felt confident that they could address challenges independently by using information available online for troubleshooting.

If my phone isn’t working then I’ll go to the computer and Google it, you know, Google’s our best friend and YouTube. You know, if there’s any videos on how to do something to and if all else fails, then I’ll call customer support. But I always try to figure it out on my own.

-Female, 62 years

In situations where participants were not able to solve a technical problem on their own, they would get support and help from younger family members.

I’m fairly confident, you know, on a computer, but I am not a tech whiz. My son-in-law is very high up in [tech company] and so I sort of rely on him for help… I don’t have any major problems, but I find some of it a little confusing because, you know, I’m not the tech generation, so I can ask him.

-Male, 75 years

One participant noted that it’s particularly important for older adults to adapt to newer technologies to help improve their lives; believing that willingness to learn about technology can open many doors for older adults.

Yeah, if I if you were to ask me…as much as sometimes, I think technology is too time consuming as far as social media stuff and everything. But for the business part of your life, the finances, I think it’s a good opportunity if you’re willing to learn how to do the online type things and stuff. Because it’s coming whether we want it to or not.

-Female, 68 years

#### External Facilitators

External facilitators are factors beyond the participant that help facilitate technology use, like technology infrastructure and easy access to health information. Participants described engaging with online tools to understand more about their health. Many participants described the internet being their first line of health information, with information elicited from internet searches informing next steps on how to approach a health issue. The participants who used mobile applications or used wearable devices (e.g. Fitbit^®^, Garmin^®^) to track their fitness described health behavior changes fueled by the health data from these apps/devices.

Participants spoke about their ability to manage their care through the health system’s EHRpatient portal. Participants emphasized the ease of use of the mobile patient portal application and how they leverage portal functionalities to improve their health care experience. Participants also expressed satisfaction with using the patient portal to communicate with their provider and/or care team.

##### Leveraging internet to learn more about their health

Participants noted that their first line of information about their health was the internet. They described using websites to understand health-related concerns and discern if it is worth escalating to a provider.

So if there’s something going on that I can’t identify, I will go online. And I know people say “Do not look up WebMD, do not look online.” But I am not looking for the worst possible thing. I just will go online and see what does this look like? What does this sound like? And gather as much information and then either try to fix it or if I can’t fix it, then I’ll get in touch with the doctor.

-Female, 67 years

Participants emphasized that when they do look up health information online, they are mindful of the source of information, seeking credible sources of information.

##### Interviewer: Which sources do you trust?

Participant: Yeah, yeah. Mayo Clinic. Cleveland Clinic. Major universities, if they have something that comes up in my Google search.

-Male, 74 years

One participant noted that while the internet can be a powerful tool to learn about their health, they are aware that information from the internet has the potential to spark more health anxiety.

I’ll Google it, but I find that it can be nothing to death, you know. It could be this, but you could be dying… I know, so I’ll still Google it. But I don’t like to take necessarily the word because I feel like, you know, like my lower back pain. You start thinking it could be my kidneys.

-Female, 69 years

##### Use of digital devices to stay active

Participants used a variety of digital health tools to track their health, including the Fitbit^®^, Garmin^®^, Virtual Reality (VR) Headsets, and mobile applications. Participants described using these tools for various things: tracking their nutrition, food intake, exercise, heart rate and sleep. One participant describes how helpful it is to have access to this data to see how their fitness has improved over time:

I use MapMyWalk. I turned it on when I went out to mow the grass yesterday in my yard, I walked just over half a mile in 20 minutes. …. I’ve been able to track the same roughly quarter mile walk in the last two weeks from almost 30 minutes. But it hasn’t felt like I’ve speed up, but it shows that I have. I like having that app to show me where I’ve been. Let me go back and look at not only where I walked, but how I’m progressing.

-Male, 71 years

Participants described leveraging the health data from their devices and applications as reinforcement to continue engaging in healthy behaviors.

When you look at your VO2 Max on your on the [Garmin^®^] watch itself, but even more in depth on the app, it’ll give you kind of trending to see if you’re going up, down staying the same. It gives you stats like while you’re in, you know, based on your sex and age, you’re in the, you know, X percent top 25% for your age group. In terms of what your current VO2 Max is. There’s some instant gratification when you move from a 43 to a 44 like I did last week, I guess, right? And so that that actually provides quantifiable reinforcement to make me want to go for that run.

-Male, 51 years

For some participants, the health data from their devices provides an impetus for them to change their health behaviors:

And with the step count [on a Fitbit^®^], just knowing how much you’re walking makes you want to just to be competitive. And we want to walk more. So I think that that was good for me.

-Female, 67 years

“Well, I try and get to that goal [step count] every day. It’s very rare that I don’t, but and if I look at it and it’s 7:00 at night and I’m way off, I’ll start walking around my house. There’s only couple times I haven’t done that. Do I wanna make that goal every day? Yeah. And then if I get over the goal two times over the goal or three times over the goal, I’m happy with myself.”

-Female, 69 years

One participant noted using their Fitbit^®^ as a way to collect health data to bring back to their PCP, prompting conversations during routine visits.

I’ve used it to communicate to her [PCP] ‘cause there was one time it [Fitbit^®^] showed my heart rate below 45 twice, and then it showed it a couple more times. But it’s so intermittent, my PCP said. Just note the date and time as long it doesn’t become all the time.

-Female, 69 years

##### Ease of use of EHR patient portal to manage care

Participants described comfort with using the patient portal to manage their care, ranging from ordering prescriptions, scheduling appointments, and communicating with their care team.

My doctor said, “You can see these results if you go download this app” and I did and it was, it’s been perfect. I’ve used it for paying bills. I’ve used it for checking results. I used it for checking my appointments. And whoa, shoot, “What do I got coming up?” You know, those type of things so yeah…it’s made it easier. With results, I can reflect back on it if I need to, or I can look again if I need to.

-Female, 70 years

One participant described a preference for using the mobile application for the health system’s patient portal because it allowed him to seamlessly integrate his healthcare management without disrupting his daily activities.

If I’m out working in the woods here, which I frequently am, I can kind of stay on top of stuff [with the MyChart mobile application] without having to take off my gloves and take off my hat and talk on the phone for a while

-Male, 68 years

Participants also noted that they feel confident in messaging their providers or care team via the patient portal to troubleshoot health issues and improve their overall healthcare experience.

If I have a kind of a nagging symptom that I’ll sometimes ask. Is this something you’d like to see me for? Do you have some other suggestion? And I’ll often get either a written message back, or sometimes we’ll arrange a phone call so they can ask a bunch more questions and help narrow things down. I’ve done that both with the with my primary care office, and occasionally with the consulting nurse as well

-Male, 68 years

### Barriers to use of technology and digital health

#### Internal Barriers

Internal barriers are intrinsic factors, such as psychological or emotional factors, that hinder the use of technology and digital health. Despite consistent use and comfort with technology and digital devices, participants had a nuanced perception of and complicated relationship with technology.

##### Emotional response to technology

Participants described mixed feelings about the impacts of technology on their lives, acknowledging the benefits of technology in improving ease of daily life, but emphasizing the negative impacts technology may have on society.

“It’s positive and it’s wonderful. In many aspects. But it is so heavily used. My feeling is that it has, you know, the whole idea, you know, bring people together, you know, social media and all… Well, if we go back to, the thing is, like Buddhism, you have to be present for a person and it’s hard to be present when you walk into a room and everybody has their phone stuck in their face and so. …You know you cannot be present for another person if you’re being distracted. And so in that aspect of it, I have very negative feelings about it.”

-Male, 75 years

In a few instances, participants described feeling burnt out by the overconsumption of digital content, even going as far as saying that technology may be the downfall of society.

“But I think that’s where we’re, I don’t know, different. I think computers are the downfall of all of us, to be honest.”

-Female, 70 years

“I mean, frankly, I’m burned out on it. Really burned out on it. I would, I would really like to just be able to throw my cell phone and my laptop in a recycled trash you know.”

-Male, 69 years

##### Negative impact on social connections

Participants described at length the impact of technology on social connections, with participants believing that technology has the ability to isolate individuals and impede meaningful social interactions. Some participants described how the rise of text messaging and social media makes it harder to have live conversations with loved ones:

“So I point to like how people are like allergic to talking on the phone, right? Like if you call me, I’m gonna wonder why and probably reject the call and wait for you to text me what you want. And I think that’s pretty prevalent as well. And I think that is just another indicator of kind of where we’re headed, right? And so the more technology we have, the more disconnected we become.”

-Male, 51 years

“It’s like what my friends do nothing but text. They do not take phone calls, do not do phone calls. I miss that. I like hearing voices, you know?”

-Female, 69 years

Another participant described how people are more willing to hide behind technology to fracture social connections.

“It’s just that to me, so much has changed because of technology and it’s not positive change, I don’t think. I think you’re more apt if you’re upset about something, just, on your computer and exclamation and cap locks and, you know, say things that you normally would not, and it’s allowed people to step over the line with each other. It just has. I’ve done it.”

-Female, 70 years

Lastly, one participant explicitly noted feeling extremely isolated because of technology use in modern day.

“I just feel like it makes my life…I feel as if electronic devices have completely isolated us. And I feel extremely isolated at this point in my life, so. Yeah. It’s just, it’s a double-edged sword.”

-Male, 69 years

#### External Barriers

External barriers to digital health and technology use are those that arise from the outside environment which impact participants’ ability and willingness to engage with digital health and technology. A significant majority of our participants described fears of breaches to data privacy, security and being victims of internet scams as a major deterrent from engaging with technology.

##### Fear of scamming and data privacy

Most notable in our findings was the fear of technology being used for scams, data breaches and identity theft. Many participants described being aware of scams using text messages or the internet, and a handful of participants described firsthand experiences of being scammed.

I have [gotten scammed]. Yeah, I had a couple of guys call me claiming they were police officers and that I needed to go down to the local courthouse here today or be arrested or something like that, unless I gave him so much money per day for something and they haven’t gone at first. And then I realized it was, you know, they were tag teaming me.

-Male, 61 years

I get scammed constantly…yeah, it’s just, yeah, it’s absurd how much there is and I’ve lost some money. I mean, I at one point I lost…I lost about 1400 bucks of a scam. A crypto scam.

-Male, 69 years

One participant noted how their experience being scammed has eroded trust in technology, making them weary of use.

People have hacked my credit card because I, you know, ordered some things online and things like that. So yeah, no, I don’t trust [technology] at all.

-Female, 65 years

Due to their own (or their friends/family’s) previous experiences being scammed, participants expressed seeking out information to protect themselves, knowing that scammers often target older people.

“I hear it on TV. And today they were talking about identity theft at 5:45 this morning. And what to do and what type of emails to look out for and stuff? I listen to it because I want to see if there’s anything new I can learn, you know”

-Female, 69 years

And I think it’s the really scary…even on the phone, a lot of older people get scammed. You know the phone calls. I don’t usually answer the phone if it’s not a number I recognize, and I know if it’s somebody that’s trying to get ahold of me, they’ll leave me a message.

-Female, 68 years

Alongside being weary of scams, participants described an overall distrust of information online.

“I don’t really trust anything that comes from, like on Facebook? If it’s not coming from somebody that I know well, and even then stuff on Facebook, I’ll sometimes Google it or look it up to try and figure out does that, could that be real?”

-Male, 74 years

Importantly, participants called out being wary of weblinks, irrespective of who the sender is of the links.

“I’m not in the habit like when I’m texting and stuff, people will text me links and things I won’t do it. I won’t click on it. If they have a phone number and address or something, they want me to look at, then you know I go to them or they go to me or something, like I don’t click on anything through text.”

-Male, 61 years

“I don’t click on any links or anything. I’ve had two friends click on links and believe them and lost thousands and 10s of thousands of dollars.”

-Female, 69 years

###### Perceptions of the Patient Voices Video as a digital health tool

The secondary objective of this study sought to understand if older adults found the *Patient Voices Video* to be an acceptable digital health tool for lung cancer screening. When shown the *Patient Voices Video*, participants emphasized that it has the potential to be a valuable digital health communication to educate patients about lung cancer screening, but also noted that it would benefit from a few design and delivery modifications.

### Overall approval of content and packaging of video

Participants expressed positive feelings about the content and key messaging of the video. In one instance, a participant noted that the video did not perpetuate smoking-related stigma when providing information about returning for on time lung cancer screening.

Well, I think I liked it. It was positive information. Because it didn’t make you feel bad for smoking. It just said we need to watch you. We need to do a little extra for you. Even if you’ve quit 15 years ago, I noticed that and things like that. So I think that’s it, but it was done in a very positive manner. Instead of frightening somebody into doing something. But I liked it.

Female, 70 years

Another participant noted that the video has the potential to relieve any anxiety pre-scan by providing the appropriate amount of information prior to LCS.

I’d be like, ‘Oh yeah, I have this, this, this lung screening that I’m kind of scared about. I’m gonna take [my doctor’s] advice and watch this video and get more information, so I’m not so fearful.’

-Female, 55 years

### Preferences for patient portal as the video delivery platform

Participants across age ranges described a preference for receiving any cancer screening related content via the patient portal.

Participant: And then would that video get pushed to them or would they have to go into the portal and actually find it?

Interviewer: Yeah, they’ll get a notification that they have a MyChart message.

Participant: In that case, yeah, I totally click on that and watch it.

-Male, 51 years

I prefer something through my patient portal. I get so much one thing that [health system] does do is they send way too much mail out…So I would much rather receive an e-mail or something of that sort, or text message, anything like that, and I would probably be more apt to check that way than I would through mail, so yeah.

-Female, 70 years

### Improvements to the delivery of video

While the content and messaging of the video was found to be largely appropriate, participants also emphasized that sending the video via a web link may appear suspicious to older adults.

If an older person like me, you get even if you get sent a link through a secure portal, you’re still hesitant to click on a link. I always am.

-Female, 69 years

A way this could be mitigated was by emphasizing the credibility of this digital health communication, potentially by adding references to peer-reviewed sources.

The other thing that I thought was maybe missing is the authorities for all this. There’s no citation to, you know, the National Institutes of Health, although those are going to be less useful in the future I suppose, unfortunately, but I mean major, you know, the major medical organizations. There’s no authority for the statements that are being made in the in the video.

-Male, 74 years

The solution to enhance credibility may be even as simple as adding extra language to assure the security of the weblink to the *Patient Voices Video*.

You might, you know, say below is a link to a video we’d like you to watch about low dose CT scans and we assure you that this link is secure. Just add a few words you know.

-Female, 69 years

## Discussion

This study aimed to uncover older adults’ attitudes and perceptions on digital health and technology, using the *Patient Voices Video* LCS screening communication tool as a case study. Overall, we found that older adults overwhelmingly expressed comfort and self-efficacy with technology and described leveraging digital tools to improve their health care, such as wearable devices, mobile apps, and the patient portal. However, participants noted significant concerns about data privacy and security that inhibit their willingness to engage in technology, which could impact their willingness to engage in digital health tools. When shown the *Patient Voices Video*, participants highlighted the need for additional security and credibility when clicking a video link, despite being delivered via patient portal from their healthcare system. This feedback underscores the importance of developing digital health content that is credible, informative and well-packaged to optimize engagement by older adults. Results of our study were guided by CFIR and categorized as internal or external facilitators and barriers to delineate areas of intervention.

Internal facilitators provide important context to better understand engagement and perceptions of digital health among older adults. We found that the two main internal facilitators to technology use were comfort with technology and self-efficacy in troubleshooting issues. While previous studies have found that older adults are slower adopters of technology and digital tools compared to their younger counterparts [7, 8, 24], many of these studies were conducted prior to the COVID-19 pandemic and do not reflect the current level of technology adoption among older adults, which drastically shifted after 2020. More recent research has found that use of technology in older adults has increased dramatically in the last decade [6, 25]. Our findings support the growing body of literature showing that older adults are capable and willing to adopt digital tools for their health care and management. A 2022 report from Pew Research Center noted that around 61% adults ages 65 and older report owning smartphones, a marked increase from 46% in 2018, and 13% in 2012 [26]. In our study, we also found an explicit preference for smartphones over desktop computers by participants. As we see people moving away from desktop computers, digital health tools must be designed to be optimized for mobile devices to ensure maximum engagement.

While it is harder to address internal barriers from an intervention perspective, these factors are important to consider when evaluating engagement with digital health interventions. Our study found that older adults expressed fears of social isolation which are exacerbated by living in the technological age. This finding contrasts with other research, which has largely reported that older adults use digital technology to improve social wellbeing and connectedness [27–29]. Given the fears of social isolation, coupled with emotional response of burn out related to technology, older adults may be hesitant to engage with additional digital tools despite reporting feeling comfortable and able to use these tools. Therefore, we may see decreased in engagement with digital tools which are entirely unrelated to capacity and ability to use technology.

In contrast to internal barriers like social isolation and emotional response, external barriers outline areas of action or intervention in improving digital tools. The most significant external barrier that was reported in our study was the fear related to data privacy and security. This finding is notable in the current context of overall digital distrust among older adults. A 2023 systematic review on older adults’ attitudes toward technology noted that older adults place high value on their privacy, are conservative about data sharing, and are fearful of misuse of personal data by external organizations [30]. This, coupled with the fact that digital distrust can lead older adults to resist digital health services [31] underscores the importance of addressing these concerns when developing digital health tools. When shown the *Patient Voices Video*, we observed privacy concerns from some participants, who mentioned they may be wary of engaging with a digital tool if their provider did not explicitly confirm its safety. Thus, when developing digital health tools for older adults, it’s important for health systems to consider how to improve trust with the intervention recipients. Ongoing, bidirectional informational exchanges with patients can improve trust in digital health research, as well as engaging patients in the design and development of digital health interventions [32].

External facilitators are tools that researchers can leverage to improve engagement with digital health tools. In our study, participants noted that the patient portal facilitated easy management of their health, including scheduling appointments or ordering prescriptions. When asked about the delivery of the *Patient Voices Video*, participants noted relying heavily on the patient portal for reminders, with a preference of getting important communication through that platform. Older adults use the patient portal extensively and have reported feeling comfortable using the patient portal in other contexts [33, 34], so the patient portal may be a successful platform to deliver digital health interventions. We also found that physical activity prompted by wearable devices and mobile applications were facilitators to engaging with digital health. While studies utilizing wearable devices and mobile health applications have routinely excluded older adults [35], this finding emphasizes that studies testing digital health interventions using wearables or mobile applications can and should include older adults.

This qualitative study has some limitations, which provide opportunities for future research. Firstly, all participants had or have had health insurance (including commercial insurance and/or Medicare) and participated in LCS at least once. Our study sample may demonstrate a higher level of self-efficacy than those who have not participated in LCS. Secondly, interviews were primarily offered by Microsoft Teams and subsequently offered via telephone if they were unable to participate via Teams. Individuals who have more extensive barriers to technology use or extremely high levels of distrust were likely not captured in our sample. These individuals may provide unique perspectives that may differ than what was captured in our study. More research may be warranted to explore perceptions of digital health and technology among those with lower levels of health literacy. Third, generalizability of findings is limited by use of a single study population in a setting of insured adults eligible for LCS in the Pacific Northwest which may not reflect the diversity of U.S. healthcare settings, particularly those with high proportion of people who are uninsured. As of 2022, members of KPWA resemble the overall population of Washington in various sociodemographic factors, with the exception that KPWA membership includes a higher proportion of White members compared to the overall population in WA [36]. Similar studies may be warranted in different healthcare settings, specifically health systems that have populations that are more diverse by race/ethnicity and insurance type. Overall study strengths included a robust rapid analysis method, which employed five analysts to ensure results were not biased by a single analyst. Further, the use of the updated CFIR across the interview guide, coding, analysis and presentation of results allowed for an in-depth exploration of barriers to adoption of an intervention across multiple domains of the framework, providing actionable insights for improving delivery and design of digital health interventions for this population.

## Conclusions

Older adults showed high levels of technology use and leveraged various digital tools (such as wearable devices, mobile applications, and patient portals) to manage their care. Despite demonstrating high levels of tech literacy, technology use may be inhibited in the older adult population due to emotional response to technology, impact on social connections and data privacy concerns. When developing digital health tools for this population, we must consider these factors in design and implementation of interventions.

## Supplementary Material

Supplementary Files

This is a list of supplementary files associated with this preprint. Click to download.

- AppendixInterviewGuide.docx

## Figures and Tables

**Table 1:** Sample interview guide questions informed by CFIR constructs

Construct	Definition (in this study context)	Sample Corresponding Interview Questions
**V. C. Assessing Context**	Collect information to identify and appraise barriers and facilitators to using technology/digital health	*Tell me a little bit about your use of technology in your day-to-day life*. *Have you ever used digital health in your healthcare?*
**IV. A. Need**	The degree to which the patient has healthcare needs that will be addressed by digital health	*What are the pros of using the patient portal? What are the cons?*
**IV. B. Capability**	The degree to which the patient has competence, knowledge and skills to use digital health/technology	*Do you normally use technology on your own, meaning without anyone helping you? What are some challenges you encounter?* *How comfortable do you feel using the patient portal?*
**V.B.2. Assessing Needs of Innovation Recipients**	Collect information about the priorities, preferences, and needs of patients to guide implementation and delivery of the innovation	*Describe to me what you use the patient portal for*. *What are some ways the patient portal can be improved to work better for you?*
**I. G. Innovation Design**	The innovation is well designed and packaged, including how it is assembled, bundled, and presented	*Now that you’ve watched the video, what’s your reaction? What did you like or not like about the experience?*

**Table 2:** RAP-GAP Analysis Steps Outlined by Hsu et al. [23] as applied in this study

RAP-GAP Step	Application
1. Plan and prepare	RAP-GAP lead identified a 5-person research team with qualitative experience All members were trained with information sheets created by Hsu et al. Pre-structured excel workbooks created with selected domains
2. Engage with data individually	Research team members independently reviewed and coded 2–4 transcripts Research team members populated pre-structured excel workbooks
3. Engage with data as a group	RAP-GAP team lead uploaded all the coded insights to a virtual white board 90-minute group session: collaborative process where insights were grouped together within domains to generate findings and emergent themes
4. Collate learnings	RAP-GAP lead exported all insights, quotes and themes from Steps 2 and 3 to create a master analytic dataset
5. Summarize findings	RAP-GAP lead created a coding memo compiling all major findings and circulated it to the RAP-GAP participants for confirmation and validation

## Data Availability

The datasets used and/or analyzed during the current study are available from the corresponding author on reasonable request.

## Abbreviations

LCS: Lung cancer screening
PCP: primary care physician
KPWA: Kaiser Permanente Washington
LDCT: Low-dose computed tomography
EHR: Electronic health record
CFIR: Consolidated Framework for Implementation Research
PI: Principal investigator
PAB: Patient advisory board
Rap-GAP: Rapid group analysis process

## References

[1] Surveillance Research Program, National Cancer Institute. SEER. 2025. seer.cancer.gov/seerstat. Accessed 10 Jul 2025.

[2] Ronquillo Y, Meyers A, Korvek SJ. Digital Health. StatPearls. Treasure Island. (FL): StatPearls Publishing; 2025.

[3] Parikh RB, Basen-Enquist KM, Bradley C, Estrin D, Levy M, Lichtenfeld JL, Digital Health Applications in Oncology: An Opportunity to Seize. J Natl Cancer Inst. 2022;114:1338–9. 10.1093/jnci/djac108. 35640986 PMC9384132

[4] Chow R, Drkulec H, Im JHB, Tsai J, Nafees A, Kumar S, The Use of Wearable Devices in Oncology Patients: A Systematic Review. Oncologist. 2024;29:e419–30. 10.1093/oncolo/oyad305. 37971410 PMC10994271

[5] Dreher N, Hadeler EK, Hartman SJ, Wong EC, Acerbi I, Rugo HS, Fitbit Usage in Patients With Breast Cancer Undergoing Chemotherapy. Clin Breast Cancer. 2019;19:443–e4491. 10.1016/j.clbc.2019.05.005. 31285177

[6] Mace RA, Mattos MK, Vranceanu A-M. Older adults can use technology: why healthcare professionals must overcome ageism in digital health. Transl Behav Med. 2022;12:1102–5. 10.1093/tbm/ibac070. 36073770 PMC9494377

[7] Kuerbis A, Mulliken A, Muench F, Moore A, Gardner A. Older adults and mobile technology: Factors that enhance and inhibit utilization in the context of behavioral health. Ment Health Addict Res. 2017;2. 10.15761/MHAR.1000136.

[8] Gell NM, Rosenberg DE, Demiris G, LaCroix AZ, Patel KV. Patterns of technology use among older adults with and without disabilities. Gerontologist. 2015;55:412–21. 10.1093/geront/gnt166. 24379019 PMC4542705

[9] Anthony DL, Campos-Castillo C, Lim PS. Who Isn’t Using Patient Portals And Why? Evidence And Implications From A National Sample Of US Adults. Health Aff (Millwood). 2018;37:1948–54. 10.1377/hlthaff.2018.05117. 30633673

[10] Breen KE, Tuman M, Bertelsen CE, Sheehan M, Wylie D, Fleischut MH, Factors Influencing Patient Preferences for Telehealth Cancer Genetic Counseling During the COVID-19 Pandemic. JCO Oncol Pract. 2022;18:e462–71. 10.1200/OP.21.00301. 34652959 PMC9014422

[11] Yang R, Zeng K, Jiang Y, Prevalence. Factors, and Association of Electronic Communication Use With Patient-Perceived Quality of Care From the 2019 Health Information National Trends Survey 5-Cycle 3: Exploratory Study. J Med Internet Res. 2022;24:e27167. 10.2196/27167. 35119369 PMC8857700

[12] Sixsmith A, Horst BR, Simeonov D, Mihailidis A. Older People’s Use of Digital Technology During the COVID-19 Pandemic. Bull Sci Technol Soc. 2022;42:19–24. 10.1177/02704676221094731. 38603230 PMC9038938

[13] Garcia Reyes EP, Kelly R, Buchanan G, Waycott J. Understanding Older Adults’ Experiences With Technologies for Health Self-management: Interview Study. JMIR Aging. 2023;6:e43197. 10.2196/43197. 36943333 PMC10131633

[14] Knotnerus HR, Ngo HTN, Maarsingh OR, van Vugt VA. Understanding Older Adults’ Experiences With a Digital Health Platform in General Practice: Qualitative Interview Study. JMIR Aging. 2024;7:e59168. 10.2196/59168. 39212599 PMC11378695

[15] Cancer Stat Facts. Lung and Bronchus Cancer. SEER. https://seer.cancer.gov/statfacts/html/lungb.html. Accessed 7 Feb 2023.

[16] Blake KD, Thai C, Falisi A, Chou W-YS, Oh A, Jackson D, Video-Based Interventions for Cancer Control: A Systematic Review. Health Educ Behav. 2020;47:249–57. 10.1177/1090198119887210. 31701780

[17] Odole IP, Andersen M, Richman IB. Digital Interventions to Support Lung Cancer Screening: A Systematic Review. Am J Prev Med. 2024;66:899–908. 10.1016/j.amepre.2024.01.007. 38246408 PMC11451259

[18] Luce C, Palazzo L, Anderson ML, Carter-Bawa L, Gao H, Green BB, A pragmatic randomized clinical trial of multilevel interventions to improve adherence to lung cancer screening (The Larch Study): Study protocol. Contemp Clin Trials. 2024;140:107495. 10.1016/j.cct.2024.107495. 38467273 PMC11065591

[19] About Kaiser Permanente Washington. Kaiser Permanente. 2024. https://wa.kaiserpermanente.org/html/public/about

[20] Lung, Cancer. Screening. U.S. Preventative Services Task Force. 2021. https://www.uspreventiveservicestaskforce.org/uspstf/recommendation/lung-cancer-screening

[21] Damschroder LJ, Reardon CM, Widerquist MAO, Lowery J. The updated Consolidated Framework for Implementation Research based on user feedback. Implement Sci. 2022;17:75. 10.1186/s13012-022-01245-0. 36309746 PMC9617234

[22] The COM-B Model for Behavior Change. The Decision Lab. https://thedecisionlab.com/reference-guide/organizational-behavior/the-com-b-model-for-behavior-change

[23] Hsu C, Mogk J, Hansell L, Glass JE, Allen C. Rapid Group Analysis Process (Rap-GAP): A Novel Approach to Expedite Qualitative Health Research Data Analysis. Int J Qualitative Methods. 2024;23. 10.1177/16094069241256275. PMC12753008 41477558

[24] Poli A, Kelfve S, Klompstra L, Strömberg A, Jaarsma T, Motel-Klingebiel A. Prediction of (Non)Participation of Older People in Digital Health Research: Exergame Intervention Study. J Med Internet Res. 2020;22:e17884. 10.2196/17884. 32501275 PMC7305561

[25] Mobile Fact Sheet: Tech Adoption Trends. Pew Research Center. 2024. https://www.pewresearch.org/internet/fact-sheet/mobile/. Accessed 2 Mar 2024.

[26] Faveiro M. Share of those 65 and older who are tech users has grown in the past decade. Short Reads. Washington, DC: Pew Research Center; 2022.

[27] Balki E, Hayes N, Holland C. Effectiveness of Technology Interventions in Addressing Social Isolation, Connectedness, and Loneliness in Older Adults: Systematic Umbrella Review. JMIR Aging. 2022;5:e40125. 10.2196/40125. 36279155 PMC9641519

[28] Sen K, Prybutok G, Prybutok V. The use of digital technology for social wellbeing reduces social isolation in older adults: A systematic review. SSM Popul Health. 2022;17:101020. 10.1016/j.ssmph.2021.101020. 35024424 PMC8733322

[29] Umoh ME, Prichett L, Boyd CM, Cudjoe TKM. Impact of technology on social isolation: Longitudinal analysis from the National Health Aging Trends Study. J Am Geriatr Soc. 2023;71:1117–23. 10.1111/jgs.18179. 36519748 PMC10089961

[30] Zhang M. Older people’s attitudes towards emerging technologies: A systematic literature review. Public Underst Sci. 2023;32:948–68. 10.1177/09636625231171677. 37204075 PMC10631270

[31] Ezeudoka BC, Fan M. Exploring the impact of digital distrust on user resistance to e-health services among older adults: the moderating effect of anticipated regret. Humanit Soc Sci Commun. 2024;11:1190. 10.1057/s41599-024-03457-9.

[32] Vitak J, Shilton K, Trust, Accessibility Considerations When Conducting Mobile Technologies Research With Older Adults. Privacy and Security, and. National Academies of Sciences, Engineering, and Medicine; Division of Behavioral and Social Sciences and Education; Board on Behavioral, Cognitive, and Sensory Sciences. Washington (DC): National Academies Press (US); 2020.

[33] Anthony D. Use and Experiences with Patient Portals Among Older Adults: National Poll on Healthy Aging. University of Michigan Institute for Healthcare Policy and Innovation; 2023.

[34] Nahm E-S, Sagherian K, Zhu S. Use of Patient Portals in Older Adults: A Comparison of Three Samples. Stud Health Technol Inf. 2016;225:354–8. 27332221

[35] Guu T-W, Muurling M, Khan Z, Kalafatis C, Aarsland D, Ffytche D, Wearable devices: underrepresentation in the ageing society. Lancet Digit Health. 2023;5:e336–7. 10.1016/S2589-7500(23)00069-9. 37236695

[36] Davis AC, Voelkel JL, Remmers CL, Adams JL, McGlynn EA. Comparing Kaiser Permanente Members to the General Population: Implications for Generalizability of Research. TPJ. 2023;27:87–98. 10.7812/TPP/22.172. PMC10266863 37170584

